# Building Trauma and EMS Systems Capacity in Rwanda: Lessons and Recommendations

**DOI:** 10.5334/aogh.3324

**Published:** 2021-10-26

**Authors:** Sudha Jayaraman, Faustin Ntirenganya, Menelas Nkeshimana, Ashley Rosenberg, Theophile Dushime, Ignace Kabagema, Jean Marie Uwitonze, Eric Uwitonize, Jeanne d’Arc Nyinawankusi, Robert Riviello, Irene Bagahirwa, Kenneth L. Williams, Elizabeth Krebs, Rebecca Maine, Paulin Banguti, Stephen Rulisa, Patrick Kyamanywa, Jean Claude Byiringiro

**Affiliations:** 1 Virginia Commonwealth University, Department of Surgery, US; 2 University Teaching Hospital – Kigali, Department of Surgery, RW; 3 University Teaching Hospital – Kigali, Department of Accident and Emergency, RW; 4 Ministry of Health of Rwanda, Service d’Aide Medicale Urgente, RW; 5 Brigham and Women’s Hospital/Harvard Medical School, Department of Surgery, US; 6 Rwanda Biomedical Centre, Division of NCDs, Injury Unit, RW; 7 Thomas Jefferson University Hospital, Department of Emergency Medicine, US; 8 Harborview Medical Center, University of Washington, Department of Surgery, US; 9 University of Rwanda, Department of Anaesthesia, Emergency Medicine and Critical Care and King Faisal Hospital, RW; 10 University Teaching Hospital – Kigali, Department of Obstetrics and Gynecology, RW; 11 University of Rwanda College of Medicine and Health Sciences, RW; 12 Kampala International University, US; 13 University of Utah, Department of Surgery, US

## Abstract

**Background:**

Surgical capacity building has gained substantial momentum. However, care at the hospital level depends on improved access to emergency services. There is no established model for facilitating trauma and EMS system capacity in LMIC settings. This manuscript describes our model for multi-disciplinary collaboration to advance trauma and EMS capacity in Rwanda, along with our lessons and recommendations.

**Methods:**

After high-level meetings at the Ministry of Health in Rwanda (MOH), in 2016, a capacity building plan focusing on improved clinical services, quality improvement/research and leadership capacity across prehospital and emergency settings. The main themes for the collaborative model included for empowerment of staff, improving clinical service delivery, and investing in systems and infrastructure. Funding was sought and incorporated into the Sector Wide Approaches to Planning process at the Ministry of Health of Rwanda.

**Findings:**

A shared mental model was created through a fully funded immersion program for Rwandese leaders from emergency medicine, nursing, prehospital care, and injury policy. Prehospital care delivery was standardized within Kigali through a train-the-trainers program with four new context-appropriate short courses in trauma, medical, obstetric/neonatal, and pediatric emergencies and expanded across the country to reach >600 staff at district and provincial hospitals. Forty-two protocols and checklists were implemented to standardize prehospital care across specialties. The WHO Trauma Registry was instituted across four major referral centers in the country capturing over 5,000 injured patients. Long-term research capacity development included Masters’ Degree support for 11 staff.

**Conclusions and Recommendations:**

This collaboration was highly productive in empowering staff and leadership, standardizing clinical service delivery in EMS, and investing in systems and infrastructure. This can be a useful model for trauma and EMS system capacity development in other LMICs.

## Background

Access to surgical services world-wide is poor – an estimated five billion lack reliable surgical care with the majority of this unmet need in low-and-middle-income countries (LMICs) [1,2]. Only 3–5% of the 313 million operations performed annually occur in LMICs [3]. Trauma accounts for the majority of global surgical burden (68%) [4]. Again, LMICs bear the brunt, especially for childhood deaths from trauma [5]. Adequate emergency care could prevent over 45% of all deaths worldwide [4,6]. Trauma and emergency systems have evolved in high income settings to address this issue but are less common in LMICs.

The WHO and World Bank have encouraged trauma and emergency medical system (EMS) capacity building as being fundamental to health systems, and the UN’s Sustainable Development Goals have specifically included cutting road traffic deaths by half globally in the next decade as an important priority (SDG 3.6) [7,8]. However, there is no established model for facilitating trauma and EMS system capacity in LMIC settings and little recognition of the broad horizontal capacity development that can result from building trauma and EMS systems.

The Ministry of Health of Rwanda (MOH), having successfully reached the Millennium Development Goals (MDGs), has prioritized addressing trauma and noncommunicable diseases. Rwanda has a population of roughly 13 million people with 70 percent below 40 years of age [9]. Of all deaths, 23% and 17% are from injuries in 5–14 year and 15-49-year cohorts, respectively [10,11,12]. Recognizing the critical nature of trauma and EMS care, the MOH created Service d’Aide Medicale d’Urgence (SAMU), a public ambulance service in 2007 to provide prehospital care in Kigali, the capital city. More recently, the MOH undertook the Rwanda Human Resources for Health program, a system-wide effort to build clinical capacity, including developing the country’s first emergency medicine training program, and created health system and EMS division strategic plans for the country [13,14,15].

Starting in 2012, a small group of surgeons from University Teaching Hospital – Kigali (CHUK), the University of Rwanda, Brigham and Women’s Hospital/Harvard Medical School, and Virginia Commonwealth University conducted pilot projects on epidemiology and prehospital services to explore trauma and EMS needs in the country [16,17,18]. We quickly realized the need to work across prehospital and hospital-based setting and clinicians from various professions (physicians, nurses and anesthetists), as well as policymakers. In this manuscript, we describe the successes and challenges of our multi-disciplinary collaboration in Rwanda, along with lessons and recommendations, for partnerships interested in building trauma and EMS capacity in LMIC settings.

## Methods

A capacity building plan was developed by the main national stakeholders involved in trauma and EMS service delivery in Rwanda—MOH/SAMU, Rwanda Biomedical Centre (RBC), University of Rwanda and CHUK––and the lead external partner, Virginia Commonwealth University (VCU, previous affiliation of first author, SJ). It focused on clinical, quality improvement/research, and leadership capacity development, across prehospital and emergency settings since these are critical aspects of effective trauma and EMS systems. Meetings were held with the leadership of SAMU and CHUK Emergency Department, Director of Injuries of RBC and the Minister of Health and Minister of State (Drs Binagwaho and Ndimubanzi) to review these plans and proposed activities.

**Figure 1:**
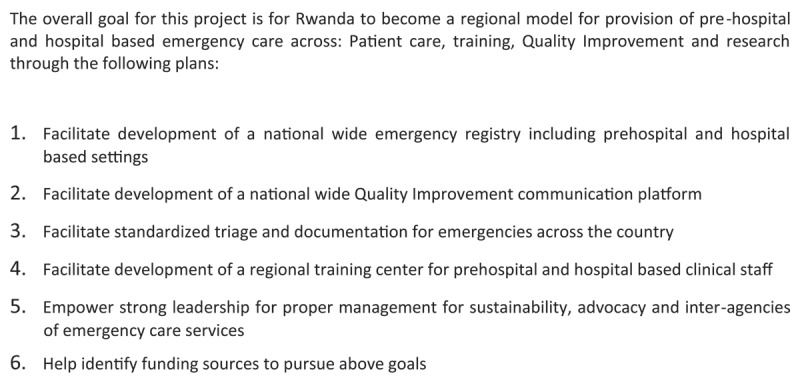
Key Aims of Memorandum of Understanding between MOH and VCU.

A memorandum of understanding between VCU and the MOH was collaboratively drafted and signed in 2017 by the Minister of Health (***Figure 1***). Key principles were local capacity building, sustainability, integration within Rwanda’s existing health systems and bilaterality, with the overall aim to become a regional model for trauma and EMS services.

The collaboration focused on three themes, modeled after Paul Farmer’s “4 Ss – Staff, Stuff, Space and Systems” [19].

1. *Create a Shared Mental Model* – Support leadership and advocacy through growth and empowerment of staff in Rwanda. Aim for leaders from different aspects of the health system in Rwanda to develop a shared mental model of trauma and EMS systems, promote their leadership locally and internationally through conference participation and empower them for advocacy efforts.
2. *Improve Clinical Service Delivery* – through investment in trauma and EMS training, standardization of care and documentation.
3. *Invest in Systems Infrastructure* – including quality improvement, research, pre-hospital, and in-hospital trauma registries and communication systems.

External funding was sought for each project, and plans and funds were integrated into the Ministry’s sector-wide approach to health system planning to ensure transparency, accountability, and adequate monitoring and evaluation. Milestones for reporting to internal and external stakeholders were based on end points of each project which were outlined as targets and deliverables at the outset. Regular internal reporting to the MOH was based on established local standards.

## Findings

The collaboration’s overall activities and productivity are summarized in ***Table 1***.

**Table 1:** Activities Supporting Trauma & EMS Capacity in Rwanda.


THEMES AND PROJECTS	ACTIVITIES	FUNDING	SUCCESSES	CHALLENGES

**Shared Mental Model**				

Leadership	Immersion in EMS, emergency medicine, trauma care, cardiac emergency care, critical care, hospital admin at VCU, state EMS systems at Virginia Department of Health Office of EMS, EMS field organization at Richmond Ambulance Authority	VCU-Crone Scholars Program; Philanthropy; VCU SOM	7 Leaders from EMS, EM, Injury Policy, MOH; 15 weeks; presentations at VCU, Virginia EMS Symposium, EMS World Expo	Retention of these leaders in Rwanda long enough to develop and implement their shared vision-> continued mentorship and support through ongoing collaboration

Advocacy	First Rwanda National Trauma Symposium	NIH NCI P20; VCU SOM	over 100 participants; results published	Potential to be one-off without ongoing momentum and support-> regular meetings to be led by RBC in partnership with stakeholders

**Clinical Service Delivery**				

Prehospital and Emergency Training Courses	4 Prehospital/Emergency Courses through train-the-trainers program for trauma, medical, pediatric, and obstetric/neonatal emergencies; Educator course for Instructor core	Rotary Foundation Global Grant	25 Instructors; > 600 trained doctors, nurses, anesthetists, midwives from provincial and district hospitals; 4 manuscripts submitted; EMS Manual for LMICs in progress	Wide scale-up and periodic refresher training needs further funding-> to be incorporated into annual SAMU outreach budget

Standardization of EMS Care through Protocols and Checklists	42 protocols and checklists created, implemented as national prehospital standard for SAMU	NIH Fogarty R21	Checklist implementation being evaluated for 6 index conditions currently	Regular QI processes to maintain quality-> support through ongoing collaboration

**Systems and Infrastructure**				

QI and Research Capacity	Short mentored research program – 4 teams; Masters’ degrees support for nurses, anesthetists, doctors from SAMU and CHUK	NIH Fogarty R21; VCU SOM	Presentations at African Congress on Emergency Medicine and College of Surgeons of East, Central and Southern Africa; 11 staff completing Masters’ degrees	Regular research projects by SAMU -> support through ongoing collaboration

Data Infrastructure – Prehospital Registry	REDCap prehospital registry 2013–2018, Google dashboard 2018-present	Harvard Postgraduate Fellowship/AAS Global Surgery Fellowship	>15,000 prehospital records captured	Ongoing use and maintenance -> led by SAMU staff

Data Infrastructure – Multi-Institutional Hospital-Based Trauma Registry	WHO Trauma registry adopted, customized, and implemented across 4 Referral hospitals in Rwanda	NIH NCI P20	>5000 trauma patient records captured in initial 12 months	Ongoing use and maintenance -> led by RBC and hospital staff with ongoing support from WHO

Emergency Communication Systems and Innovation	Private-Academic-Government collaboration with Rwanda Build; Concept design for Rwanda912 mobile health platform for emergency communications and coordination selected top 5 finalists at Toyota Mobility Ideathon and Smart Kigali Competition in 2018	VCU SOM; NIH Fogarty R21; Toyota Finalist Award; Smart Kigali Finalist	Formal Engagement from MOH; Application submitted to NIH for software development and implementation research	Funding to develop and implement -> in progress


AAS – Association for Academic Surgery, EM – Emergency Medicine, EMS – Emergency Medical Services, MOH – Ministry of Health of Rwanda, NIH – National Institutions of Health, NCI – National Cancer Institute, QI – Quality Improvement, VCU SOM – Virginia Commonwealth University School of Medicine.

### Shared Mental Model

#### Leadership and Advocacy

Unrestricted philanthropic support led to creation of the VCU-Crone Scholars program which allowed leaders from Rwanda to spend a total of 15 weeks in the United States between 2017–2019. Staff across various disciplines—anesthesia, emergency, and prehospital care as well as injury policy—learned about systems of care in a variety of fields including emergency medical services, emergency medicine, trauma care, cardiac emergency care, critical care, as well as trauma and EMS administration at hospital and state levels. They learned about dispatch, quality improvement, supply chain, and human resource aspects of the EMS system at the internationally recognized Richmond Ambulance Authority, a leading EMS agency in the United States that has won multiple national awards. The Scholars discussed state-level policy and organization of the Virginia’s EMS system in meetings with the Virginia Department of Health Office of EMS leadership, which is a rare system even within the United States. In a spirit of bilaterality, they taught their US counterparts about the challenges they would like to address in Rwanda and the strengths of the Rwandan system.

### Improve Clinical Service Delivery

#### Prehospital and Emergency Training Courses

Although the MOH has invested substantially in staff and systems since creating the SAMU prehospital ambulance service in 2007, there is no formal training program to standardize prehospital emergency care in the country. To rapidly standardize care delivery by the SAMU team, new, context-specific short courses were developed and implemented.

The courses were created collaboratively, based on international best practices including the US National Registry Emergency Medical Technician and paramedic training curricula taught by the VCU Center for Trauma and Critical Care Education Center (CTCCE), which provides training for roughly 8000 staff in Virginia every year and received the 2017 Army-Community Partnership Award from the US Pentagon in recognition of its national leadership in education. CTCCE educators, VCU clinical faculty, SAMU leadership and clinical experts from the University Teaching Hospital-Kigali (CHUK) worked together to customize the content to the Rwandan context and delivered the courses in a multi-disciplinary fashion.

These courses were conducted as Train-the-Trainer programs and led a total of 25 of the 70 SAMU nurses and anesthetists becoming instructors. Each course was taught over two days to the SAMU Instructor core who then taught multi-disciplinary staff from district and provincial hospitals across the country in two subsequent days. An educator course covering principles of adult education, Bloom’s taxonomy, effective teaching techniques, and feedback was developed and given to the instructor core as a requirement prior to conducting any of the courses independently. All instructors received mentorship to give their first independent course and received immediate individual feedback after they delivered their first course to ensure instructional quality. Pre- and post-course assessments showed statistically significant (p < 0.05) improvements in knowledge for both the SAMU Instructor core and the district and provincial hospital staff, regardless of the level of question difficulty [20,21].

#### Standardization of EMS Care through Protocols and Checklists

We had previously implemented simple interventions for standardizing prehospital trauma care delivered by SAMU in Kigali as a pilot project [18]. Based on the success of this project, local case volumes and the need for formalized prehospital standards, we developed and implemented context-appropriate protocols across a wide variety of conditions seen by SAMU.

To support quality improvement, we had previously created a prehospital registry using REDCap^®^, a secure web application for managing data (Nashville, TN). Using this registry, 42 conditions were selected for standardization by the SAMU team including emergencies due to trauma, non-communicable diseases, obstetrics, and pediatrics, since the same system needs to deliver care for all emergencies. Both a written protocol with detailed descriptions, and a checklist highlighting the key tasks for care, were created for each condition. The drafts were created by members of this collaboration, working closely with the SAMU team, who then conducted revisions, presented the materials to all SAMU staff for broad input and ultimately approved the final versions. A pocket-sized reference guide was created and distributed to all staff on SAMU ambulances with key checklists, emergency phone numbers, supplementary clinical algorithms, and user instructions for medical equipment.

### Invest in Systems Infrastructure

#### Quality Improvement and Research Capacity

To support quality improvement and operational research capacity, we created a short-term mentored research training program for four teams of SAMU nurses and anesthetists, who typically do not have regular access to research mentorship. The teams developed research questions that could be answered using the REDCap^®^ registry with analytic support from members of this collaboration. Over five months, short didactics were given on conduct of research, abstract and manuscript writing, oral presentations, and posters. All teams developed and presented original research including at the African Conference on Emergency Medicine (AfCEM) and College of Surgeons of East Central and Southern Africa (COSECSA) [21,22,23]. Long term research capacity led to 11 staff, including anesthetists, nurses, and emergency medicine physicians, obtain advanced degrees at the University of Rwanda, University of Global Health Equity and University of Johannesburg.

#### Data Infrastructure – Prehospital Registry

Based on initial assessment of injury mortality in Kigali, we recognized the value of prehospital care [16]. We had implemented a REDCap^®^ registry in 2015 and captured routinely collected prehospital data in an analyzable electronic form [17]. In 2017–2018, the newly appointed physician Division Manager for SAMU leadership transitioned to using Google drive and a new Google dashboard was created to monitor usage of checklists for six index conditions. A time series analysis of pre- and post-checklist implementation is in process using these data. Furthermore, the ambulance records were updated to include the protocols and checklists to facilitate standardized prehospital care delivery.

#### Data Infrastructure – Multi-Institutional Hospital-Based Trauma Registry

**Figure 2:**
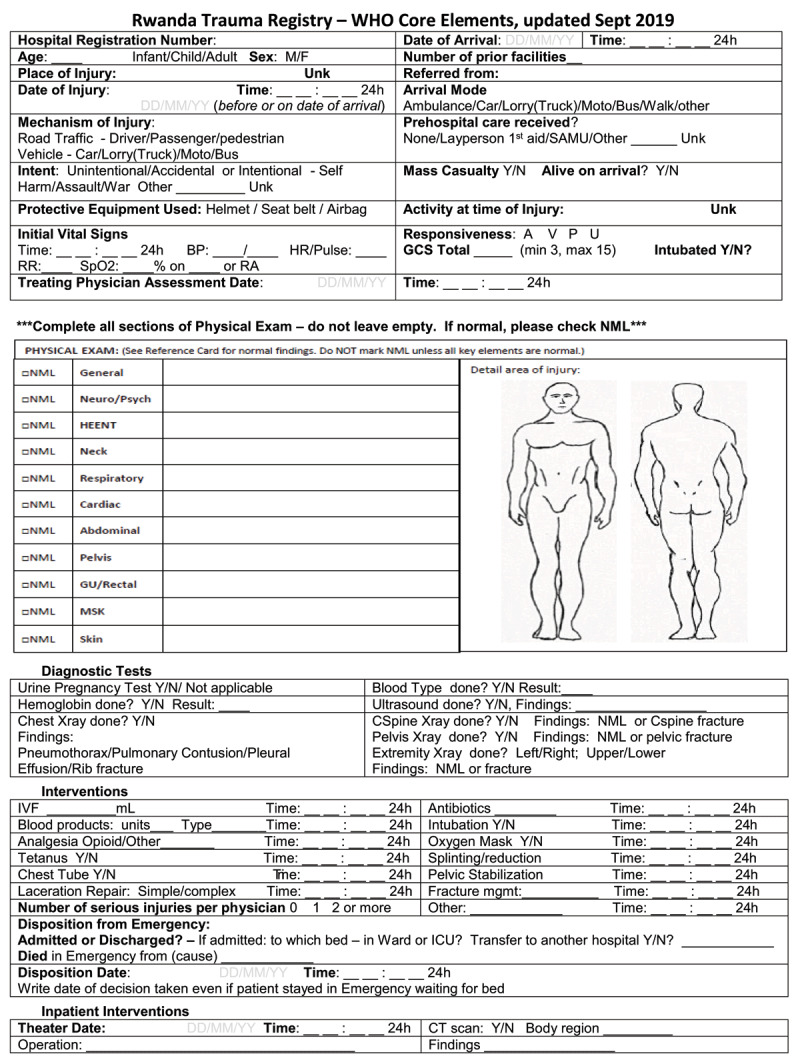
Rwanda Trauma Registry Form.

The United Nations Sustainable Development Goal (SDG) 3.6 aims to cut the number of global deaths from road traffic crashes by half by 2030 [8]. Trauma surveillance is essential to achieving this goal. We worked with the World Health Organization to implement a modified version of the new WHO Trauma Care registry in 2019. Key leaders from CHUK and the Rwanda Biomedical Centre, the implementation arm of the MOH, have led the initiative with the main objectives being facilitating surveillance, assessing resource use, supporting research, and creating local evidence to inform clinical, educational, and policy priorities in Rwanda. This registry was built on a pilot registry at CHUK and expanded to four main referral hospitals in the country and has collected over 5,000 patient encounters since implementation. It also addressed some of the data systems recommended by the 2018 Rwanda National Surgical, Obstetric, and Anesthesia plan [24]. ***Figure 2*** demonstrates the newly implemented Rwanda trauma registry form.

#### Emergency Communication Systems and Innovation

Emergency care infrastructure is an underrecognized aspect of trauma and emergency systems around the world. Hospitals, ambulances, dispatch, and the public need a unified platform for communication to ensure rapid and efficient delivery of emergency services. We identified this need in Rwanda in conjunction with key stakeholders (MOH, Rwanda National Police, CHUK Department of Accident and Emergency, SAMU, VCU, and the Rwanda Build program, a local software startup accelerator in Kigali). We have since designed an innovative context-appropriate mobile health platform which was selected as one of the top five finalists in two innovation competitions in Kigali, out of a field of 176 and 150 teams, respectively (Toyota Innovation Mobility Ideathon in 2018 and Smart Kigali competition in 2018). We also created and pilot tested new software to collect global positioning and time data from ambulances which showed that the average total prehospital time within Kigali for any emergency is 61 minutes, with 42% taking longer than 60 minutes [25,26].

## Discussion

The WHO and the World Bank have encouraged trauma and emergency system development, but no model currently exists to do so in LMICs which leaves policy makers and health system leaders without a feasible model to apply in this area. Resources and capacity for emergency care vary dramatically across settings but investing in trauma and emergency systems is likely to have broad impact for health systems. Our collaboration to facilitate trauma and emergency systems in Rwanda was highly productive across the three central themes: *creating a shared mental model, improving clinical service delivery, and investing in systems infrastructure*, and therefore may be translatable to address the need for trauma and emergency systems in other contexts. While this collaboration did not aim to result in changes in mortality or larger systems indicators through these projects, we wanted to address process and output changes that were doable and that allowed us to demonstrate the effectiveness of our partnerships. We were able to acquire funding from a wide variety of sources including United States federal grants, non-governmental foundations, academic partner institutions and philanthropy to conduct this work and based on our successes, we have been able to continue to apply for and obtain larger grants through the strength of this work despite the COVID-19 pandemic. Therefore we outline our lessons learned below to encourage others to consider them as they engage on trauma and emergency system capacity work in their settings.

### Successes, Challenges and Recommendations

*Creating a Shared Mental Model* – for clinical and policy experts, creating a shared mental model across disciplines and professional roles was essential to learn about organizational structure, quality systems, training expectations and processes of care delivery. It promoted a dialogue over the system in Rwanda from different perspectives. A key need identified was a forum to bring together clinicians, researchers, and policy makers involved in trauma work in Rwanda to collectively discuss local needs and priorities. The first Rwanda National Trauma Symposium was conducted in February 2019. Over 100 multi-disciplinary colleagues, mostly Rwandese, who provide trauma care, conduct trauma prevention and research and set policy for the country discussed the main challenges to trauma care and policy which included prehospital trauma and emergency medical systems, trauma prevention, research and policy, and resource limitations, and proposed further priorities and solutions [30]. This encouraged transparency, further priority setting and engagement of diverse local stakeholders to champion for improving trauma and emergency systems and needs to be a key first step to facilitate capacity development. Challenges include ongoing collaboration and mentorship as well as loss of momentum for regular national meetings. Despite the COVID-19 pandemic, we have conducted one virtual symposium (Nov 2020) focused on the trauma registry and including staff from multiple referral hospitals, government, academic institutions and the WHO. We have also submitted multiple grant proposals and projects with leadership roles for key stakeholders. Based on our experience, our first recommendation for other partnerships working on trauma and emergency systems development is to focus on intentionally building engagement from diverse local stakeholders. Often, collaborations narrowly focus on physicians and academics without a broader scope which limits the potential for impact. Intentionally engaging diverse stakeholders energizes the community, creates momentum to enable advocacy and allows priority-setting that is inter-professional and cross-sectoral and should be prioritized regardless of source of funding support.

*Improving Clinical Service Delivery* – The fundamental point of trauma and emergency systems are to provide high quality care at the right time. Therefore, improving emergency medical service delivery through training and standardization was our second major area of focus. The Instructor core of SAMU staff trained nearly 300 staff from district hospitals within the first year and moved on to deliver the trauma course independently and successfully to over 300 additional staff at nine hospitals around the country in the second year. The effectiveness of these courses have been published [20,21]. These courses have been integrated into the budget for standard outreach across the country by the Division of EMS for long term sustainability.

Furthermore, a total of 42 conditions due to trauma, non-communicable diseases, obstetrics, and pediatrics were selected for a written protocol with detailed descriptions, and a checklist highlighting the key tasks for care. These were approved as the prehospital standard for Kigali city with plans to validate the materials as a national prehospital standard through more extensive review across relevant professional societies in Rwanda. Leveraging this, the SAMU leadership has developed several Quality Improvement projects and long-term EMS strategies, including digitizing its data using the Health Management and Information Systems to connect pre-hospital care and in-hospital services. The training courses and protocol development also had additional benefits beyond trauma care including supply chain for medications to limit postpartum hemorrhage and other critical conditions. In next steps, we aim to facilitate training and standardization for the emergency dispatch center, biomedical and mechanical training for ambulance maintenance, Emergency Medical Technician-Basic training for ambulance drivers and emergency communications development.

Challenges include conducting regular quality improvement to ensure high quality of care is being delivered. The planned pre- and post-checklist implementation analysis was halted by COVID. However we are now in the process of comparing three time periods including post-COVID which will assess the current quality of care. Based on our results, a second key recommendation for others is to understand the existing training and care delivery systems and identify clear needs that can be addressed in an incremental fashion. A focus on building a cadre of experts and identifying ways to integrate interventions into the existing systems leads to potential for long-term sustainability.

*Investing in Systems Infrastructure* – The third focus was improving systems through quality improvement, research, and innovation. Through intensive short-term mentorship nearly 16 SAMU nurses and anesthetists, who typically do not have regular access to research mentorship, were able to develop original research and present in large fora and 11 anesthetists, nurses, and emergency medicine physicians obtained graduate degrees at the University of Rwanda, University of Global Health Equity and University of Johannesburg. At the prehospital level, a Google dashboard was implemented to track quality of care and assess effectiveness of checklist implementation. At the hospital level, the multi-site trauma registry has been expanded by the government to two more hospitals in conjunction with the WHO. It has been audited for accuracy and completeness and the quality of trauma care capabilities has been evaluated across sites to develop an in-depth understanding of the trauma care at the major referral hospitals in Rwanda [27,28,29]. Additionally, a mobile health platform to improve emergency communication in Kigali was collaboratively designed, won two innovation competitions and now has been funded by the NIH Fogarty International Center.

The Ebola outbreak in the region in 2019 and the COVID-19 pandemic since 2020 were major sources of disruption for these projects. However, the staff who have gained the critical research and quality improvement skills through this collaboration have taken on larger roles during both crises. There has been ongoing commitment by the government of Rwanda to support and expand trauma and prehospital emergency services throughout this period. A third and critical recommendation from our collaboration for others interested in improving trauma and emergency capacity is to intentionally include quality improvement, research, and innovation so that operational efficiency and effectiveness can improve. These are easily considered secondary to improving clinical service delivery but they are essential to improving the larger system which offers greater resiliency to unexpected crises based on our experience.

### Sustainability and Synergy with External Actors

At the policy level, identifying gaps and priority areas collaboratively helped our work gain momentum, transparency, and accountability. Buy-in at high levels in the MOH and multi-disciplinary approach supported effective partnerships with clear outputs. Our efforts also complemented the highly successful Human Resources for Health program by focusing on support for trauma and EMS care in Rwanda which were not fully within its scope [13,30,31]. Many of the projects have been integrated into routine functions of the MOH.

Furthermore, this collaboration’s work has led to numerous subsequent partnerships. Our work started with a focus on trauma care and epidemiology in 2012 and has since grown to a broader and multidisciplinary partnership and has led capacity across a breadth of human resources—physicians, surgeons, nurses, anesthetists, and policy makers. This multidisciplinary approach has been essential for success. All these activities raised the profile of the trauma and EMS system in Rwanda and have built health system capacity broadly across many areas, rather than just focused on one disease or process.

Additional partnerships in other clinical departments including anesthesia, emergency medicine, and critical care have grown. Through this collaboration, a VCU internal medicine physician spent a year as a Fulbright scholar at CHUK to support multidisciplinary research projects across emergency and internal medicine departments. This has now led to a feasibility trial of Vitamin C use in sepsis putting Rwanda on the cutting edge of developing locally relevant research to address a major non-communicable disease. Through a critical care collaboration through VCU and the Society of Critical Care Medicine two nurse practitioners and a pulmonary critical care physician conducted the first international Fundamentals of Critical Care short course in Kigali, Rwanda in 2018 and a train-the-trainers course in 2019. VCU and the University of Virginia have partnered to support anesthesia and critical care education in Rwanda through an observer ship program for anesthesia residents and an operational research course based on the WHO SORT-IT to develop long-term research capacity. Lastly, this work and the research capacity has developed has spurred further opportunities to collaborate with other international injury experts involved in Rwanda from the United Kingdom and larger scale grant applications for more high-impact research and capacity development. Such knock-on effects were not anticipated but clearly confirm the broad horizontal capacity development that is possible through facilitating trauma and EMS capacity building.

### Limitations

This collaboration was, by design, not the only one facilitating emergency care development in Rwanda. Local coordination across numerous external partners can be a challenge for every setting and especially in Rwanda where rapid growth and stability has drawn several international collaborations [30,31]. Having a five-year MOU allowed us to define specific plans and metrics over a set period, to avoid duplication of efforts and inefficient use of financial support. Funding is a limitation for all collaborations and this one so far has had limited but effective support from governmental, non-governmental, and philanthropic sources. Lastly, we have only tangentially involved pediatric trauma care or prevention, even though >90% of all childhood trauma deaths occurring in LMIC settings and this remains a key area of interest based on local demographics [32].

The COVID-19 pandemic has demonstrated the need for emergency and critical care services and reinforced the need for building capacity in these areas in Rwanda and around the world. The aspects of health systems that are necessary to address severe respiratory illnesses such as COVID-19 the same ones needed in obstetric emergencies, mass casualty events or cardiovascular events. Many of the activities led by this collaboration including the short courses for management of prehospital emergencies have formed a valuable foundation for responding to the pandemic. The MOH has relied on SAMU to locate, manage, and transport patients with COVID-19 to the appropriate hospitals. Many of the staff from this collaboration have applied their experiences from trauma and EMS systems capacity building to leading the country’s COVID-19 response team, further demonstrating the value of investing in trauma and EMS capacity building.

In summary, trauma and EMS system capacity building in Rwanda, based on meaningful and deliberate engagement across academic institutions, hospitals, public services, and policy makers, has been an example of horizontal capacity building. The lessons and recommendations from this collaboration’s experience in Rwanda may be useful for other LMIC leaders interested in building such systems in their settings.
